# Stable Dual miR-143 and miR-506 Upregulation Inhibits Proliferation and Cell Cycle Progression

**DOI:** 10.3390/ijms25084432

**Published:** 2024-04-17

**Authors:** Archana Shrestha, Behnaz Lahooti, A. K. M. Nawshad Hossian, Mahboubeh Madadi, Constantinos M. Mikelis, George Mattheolabakis

**Affiliations:** 1 School of Basic Pharmaceutical and Toxicological Sciences, College of Pharmacy, University of Louisiana at Monroe, Monroe, LA 71201, USA; 2 Department of Pharmaceutical Sciences, School of Pharmacy, Texas Tech University Health Sciences Center, Amarillo, TX 79106, USA; 3 Department of Marketing and Business Analytics Lucas College and Graduate School of Business, San Jose State University, San Jose, CA 95192, USA; 4 Laboratory of Molecular Pharmacology, Department of Pharmacy, University of Patras, 26504 Patras, Greece

**Keywords:** microRNA, lung cancer, lentiviral transduction, dual miR therapy, miR-143, miR-506, quantitative phase imaging

## Abstract

The mainstays of lung cancer pathogenesis are cell cycle progression dysregulation, impaired apoptosis, and unregulated cell proliferation. While individual microRNA (miR) targeting or delivering is a promising approach that has been extensively studied, combination of miR targeting can enhance therapeutic efficacy and overcome limitations present in individual miR regulations. We previously reported on the use of a miR-143 and miR-506 combination via transient transfections against lung cancer. In this study, we evaluated the effect of miR-143 and miR-506 under stable deregulations in A549 lung cancer cells. We used lentiviral transductions to either up- or downregulate the two miRs individually or in combination. The cells were sorted and analyzed for miR deregulation via qPCR. We determined the miR deregulations’ effects on the cell cycle, cell proliferation, cancer cell morphology, and cell motility. Compared to the individual miR deregulations, the combined miR upregulation demonstrated a miR-expression-dependent G2 cell cycle arrest and a significant increase in the cell doubling time, whereas the miR-143/506 dual downregulation demonstrated increased cellular motility. Furthermore, the individual miR-143 and miR-506 up- and downregulations exhibited cellular responses lacking an apparent miR-expression-dependent response in the respective analyses. Our work here indicates that, unlike the individual miR upregulations, the combinatorial miR treatment remained advantageous, even under prolonged miR upregulation. Finally, our findings demonstrate potential advantages of miR combinations vs. individual miR treatments.

## 1. Introduction

Lung and bronchus cancers are the second leading new cancer cases for both men and women and the leading causes of cancer-related deaths in the United States [1]. Nucleic acid-based therapies have attracted attention due to their capacity to target specific mechanistic responses that traditional chemotherapeutic agents are unable to target. Non-coding nucleic acid molecules have important mechanistic activities as natural cell products. Among the different types of these RNA molecules, miRNAs (miRs) are natural transcriptional products of the genome. Having 21–24 nucleotides, miRs can regulate gene expression through partial complementarity, predominantly in the 3′-UTR region of mRNAs [2]. With various diseases, such as inflammation and cancer, presenting deregulated miR expressions, these molecules represent potential tools or targets for treatment. Although their versatility allows them to regulate multiple pathways, their complicated mechanistic activities also present a challenge for the proper characterization of their targets, effects, and potential positive or negative outcomes. The miRs’ large numbers also aggravate this drawback, as several thousands of miRs have been identified [3]. Nonetheless, certain miRs have been identified as tumor suppressors, such as the miR-34 family [4], and extensive studies on such miRs have demonstrated the potential of miR-based therapeutics for cancer treatment, with activities that include apoptosis, proliferation inhibition, cell cycle regulation, and tumor microenvironment modulation, among others [4,5]. Furthermore, miRs’ versatility does not only include multiple pathway targeting, but also the fact that their utilization can expand to various cancer types. With non-small cell lung cancer (NSCLC) representing the majority of lung cancer (LC) cases [6], our lab’s major focus is the use of miRs for the inhibition of LC cell proliferation through cell cycle regulation. First discovered in 2005 by Bentwich and colleagues, miR-506 belongs to an X-chromosome-linked miR cluster which is well conserved within primate species [7]. Several studies have identified miR-506 as an anti-cancer biomolecule in various cancer types, due to its abrupt dysregulation in various cancer phenotypes [8,9]. Moreover, miR-506’s role as an anti-oncogenic miR is demonstrated by it targeting numerous biological pathways, such as through diminished epithelial–mesenchymal transition (EMT) mediated by TGF-β [10], inhibition of E-cadherin expression [11], targeting the PI3K/AKT pathway [12], and p53 response element activation [13]. miR-143’s significant downregulation has been recorded in numerous cancer types including breast [14], colorectal [15], cervical [16], ovarian [17], and non-small cell lung cancers [18]. This suggests that miR-143 can potentially act as a tumor suppressor gene for cancer regulation. Several studies have indicated the role miR-143 has in cancer regulation by suppressing various molecular oncogenic pathways, including PI3K/MAPK [19,20,21] and KRAS [14,15], and by regulation of EMT [22,23]. Besides their role in modulating numerous oncogenic pathways, both of these miRs are also reported to regulate cell cycle progression [9,18,24,25,26,27]. Similarly to traditional chemotherapeutic combinations, recently, miR combinations have attracted significant attention as well [28,29].

We recently reported on the combination of two miRs, miR-143-3p and miR-506-3p, which presented advantageous effects against NSCLC cells when transiently upregulated. It has been reported that these two miRs are individually downregulated in LC cells [9,30,31] and that they can induce apoptotic activity in LC cells, regulate CDK expression, and affect the cell cycle progression [12,24,26,27,31,32]. Our previous work [9,18,33] relied on the transient transfection of these miRs using mimics as means of a therapeutic administrations of the molecules. Although our work involved multiple types of analysis, the transient transfection can only be viewed as an acute effect or repeated acute effects/treatments of the molecules. Alternatively, stable deregulation of miRs has also been utilized to evaluate their effects in cells. This approach allows the study of miRs without the fluctuation of the miR levels that transient transfection produces. Furthermore, stable transductions can also be used to study any long-term effects of the miRs’ deregulations, especially in scenarios/diseases where long-term treatments are required. As the stably deregulated miRs can heavily impact cells, especially when the miR deregulation is associated with apoptosis, the surviving transduced cells may develop compensatory mechanisms to permit the continuation of proliferation. In such a case, cells with a stable miR deregulation may not represent cells experiencing acute (transient) miR deregulation but may resemble cells of a potential “resistant” phenotype against the respective miR deregulation.

Therefore, in this study, we stably deregulated two miRs, miR-143 and miR-506, using lentiviral transduction, and induced their respective stable up- or downregulation, individually and in combination. Our analyses indicated that the dual miR upregulation presented miR-expression-dependent advantageous behavior, including reduced proliferation and cell cycle inhibition, that the respective individual miR deregulations did not. In fact, the individual miRs alone did not present an effect that corresponded or correlated to a specific up- or downregulation of each respective miR. These results illustrate that combining multiple miRs for therapeutic purposes, i.e., miR combinations, may be advantageous compared to single miR treatments. Our study may have implications not only for the two studied miRs (i.e., miR-143 and miR-506), alone or in combination, but also for the overall understanding of how combinatorial miR treatments can be advantageous for cancer treatment.

## 2. Results

### 2.1. Stable Transduction of Cells

**Figure 1:**
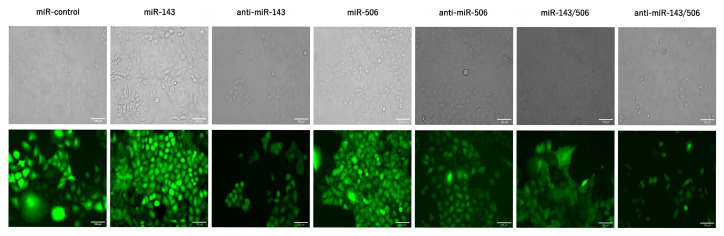
A549 cells were transduced using lentiviruses for the respective miR-143, miR-506, or dual miR-143/506 deregulations. Representative images show GFP signals from the cells as the result of the lentiviral transduction.

We stably transduced A549 cells to upregulate or downregulate miR-506 or miR-143, or perform their respective dual up-/downregulation. Our objective was to evaluate the behavior of the LC cells under constant deregulation of the miRs, which the transient transfection and its acute apoptotic activity on the cells cannot indicate. First, each cell group was transduced using lentiviruses, following the vendor’s protocol with minor adjustments. The lentivirus transduction induced GFP expression (Figure 1) and antibiotic resistance to the cells. Each transduced miR deregulation group presented puromycin (for miR-143 deregulation) or G418 (for miR-506 deregulation) resistance. The concentrations of 2.5 μg/mL for puromycin and 850 μg/mL for G418 were used to select the successfully transduced cells. Subsequently, the cells were sorted using a fluorescence-activated cell sorter (BD FACS Aria III Cell Sorter). GFP-positive cell populations were collected for subsequent analysis (Supplementary Figure S2).

We conducted cell authentication on the transduced cells to confirm the A549 cell type and to ensure the absence of any unexpected mutations or cross-contaminations. The results showed that all stably transduced cells are a 100% match to the original parental A549 cell line (Supplementary Figure S3). Finally, mycoplasma analysis ensured the cells did not present mycoplasma contamination (Supplementary Figure S4).

### 2.2. miR-143 Deregulation

#### 2.2.1. Lentiviral Transduction Induced miR-143 Deregulation

**Figure 2:**
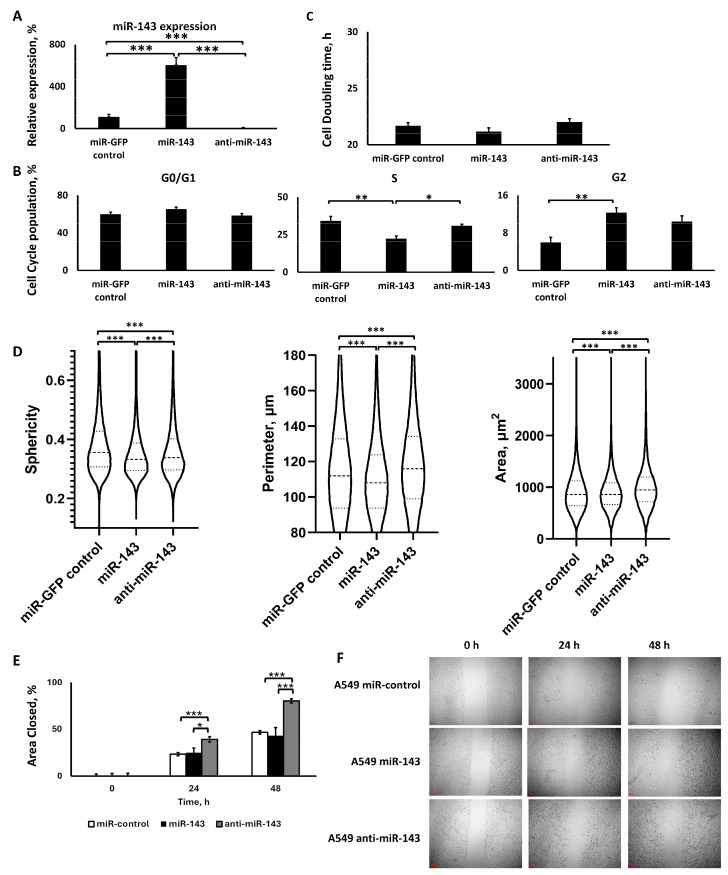
Analysis of the A549 cells transduced using lentiviruses for miR-143 up- or downregulation. (**A**) qPCR analysis of miR-143 expression; (**B**) cell cycle analysis; (**C**) cell doubling time analysis; (**D**) QPI morphometric analyses for sphericity, perimeter, and area of the cells over a 24 h period; (**E**,**F**) wound healing assay analysis (**E**) with representative images (**F**). *: *p* < 0.05, **: *p* < 0.01, ***: *p* < 0.001; one-way ANOVA followed by post hoc analysis.

We initially evaluated and confirmed the deregulation of miR-143 due to lentiviral transduction in the respective cell groups. As shown in Figure 2A, qPCR analysis with TaqMan primers, using the sorted cells that survived the lentiviral transduction and antibiotic treatment, confirmed that the lentiviral transduction upregulated or downregulated the miR-143 expression in the respective cell groups. In more detail, cells in the miR-143 upregulation group demonstrated a >6-fold increase in the levels of miR-143 compared to the miR-control group (*p* < 0.001), while the cells in the miR-143 downregulation group demonstrated a 92% decrease in miR-143 levels compared to the miR-control group (*p* < 0.001).

#### 2.2.2. miR-143 Upregulation Decreased S Phase Population, but All miR-143 Deregulations Increased G2 Phase Population, without Affecting Cell Proliferation

The cells that survived the lentiviral transduction and miR deregulation demonstrated an impacted cell cycle. Briefly, the miR-143 upregulation demonstrated a slight increase in the G0/G1 cell population by 5.4% compared to the miR-GFP control, while miR-143 downregulation demonstrated a slight 1.5% decrease in the G0/G1 cell population, though there was no statistical significance in these changes among the groups (Figure 2B). In contrast, for the S population, the miR-143 upregulation significantly decreased the cell population by 11.9% compared to the miR-control group (*p* < 0.01) and by 8.8% compared to the miR-143 downregulation group (*p* < 0.05). The miR-143 downregulation group was slightly decreased by 3.1% compared to the miR-GFP control, although there was no statistical significance between the two groups. Finally, in the G2 cell populations, the miR-143 upregulation had a significantly 2-fold larger cell population compared to the miR-GFP control (*p* < 0.01). Interestingly, the miR-143 downregulation G2 population was also 1.73-fold larger compared to the miR-GFP control group, though smaller than the miR-143 upregulation group, without achieving statistically significant differences compared to either of these groups. Thus, although the miR-143 upregulation indicated a unique change in the S population compared to the respective downregulation, this was not the case for the G2 population, where both up- and downregulation demonstrated an unexpectedly similar behavior, despite the significant opposite difference in miR expressions.

Subsequently, we analyzed the cell doubling time for the miR-143 deregulated groups and compared it to the miR-control group (Figure 2C). Our analysis did not indicate any significant differences between the groups, though there was a slight reduction by 2.3% in the miR-143 upregulation group’s doubling time and a slight increase by 1.4% in the miR-143 downregulation group’s doubling time when compared to the miR-control group, though these differences were not statistically significant (*p* larger than 0.05). 

#### 2.2.3. The Two miR-143 Deregulations Altered Cell Morphology and Motility in a Similar Manner

We utilized quantitative phase imaging (QPI) to identify three morphometric characteristics of the miR-143 deregulated cell groups and compared them to the miR-control group (Figure 2D). We monitored for changes in area, morphology, and perimeter due to miR deregulation in the A549 cells. Both miR-143 upregulation and downregulation reduced cellular sphericity, with the former inducing a 5.2% reduction and the latter inducing a 2.6% reduction, respectively, in the cells’ sphericity compared to the miR-control group. All differences were statistically significant among the groups, a result of the large number of measurements made with the methodology performed on individual cells. In contrast, the cell perimeter for miR-143 upregulation was reduced by 4.4% compared to the miR-control (*p* < 0.001), while the miR-143 downregulation induced a 3.1% (*p* < 0.001) increase in the area compared to the miR-control group and a 7.8% (*p* < 0.001) increase compared to miR-143 upregulation. Similarly, the cell area was decreased by 2% for the miR-143 upregulation group compared to the miR-143 downregulation group (*p* < 0.001), while the miR-143 downregulation was 10.1% (*p* < 0.001) higher than the control and 12.4% (*p* < 0.001) higher than the miR-143 upregulation group.

Finally, we performed a wound healing assay (Figure 2E,F, Supplementary Figure S6) to evaluate the motility of the cells and the effect of miR-143 deregulation on them. Our analysis indicated that at the 24 h time point after the formation of the wound, the miR-143 downregulation demonstrated accelerated motility compared to the miR-control and the miR-143 upregulation. In more detail, the miR-143 downregulation group demonstrated an approximately 1.6-fold larger area closed compared to the miR-control group (*p* < 0.001), and a 1.6-fold larger area closed compared to the miR-143 upregulation group (*p* < 0.05). Interestingly, miR-143 upregulation had a similar closure rate compared to the miR-control, though there were no statistically significant differences between the two groups. In contrast, for the 48 h time point after the formation of the wound, the miR-143 upregulation presented the slowest closure, but without any statistically significant difference from the control. Moreover, miR-143 downregulation demonstrated the fastest closure rate compared to the miR-control by a 1.7-fold increase (*p* < 0.001) and a 1.85-fold larger area closed compared to miR-143 upregulation (*p* < 0.001). 

### 2.3. miR-506 Deregulation

#### 2.3.1. Lentiviral Transduction Induced miR-506 Deregulation

**Figure 3:**
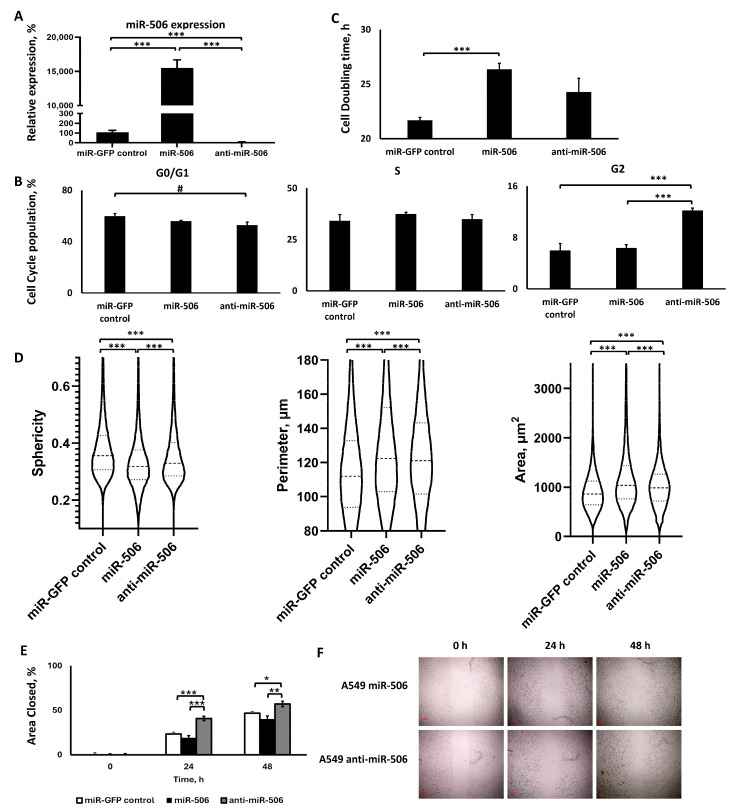
Analysis of the A549 cells transduced using lentiviruses for miR-506 up- or downregulation. (**A**) qPCR analysis of miR-506 expression; (**B**) cell cycle analysis; (**C**) cell doubling time analysis; (**D**) QPI morphometric analyses for sphericity, perimeter, and area of the cells over a 24 h period; (**E**,**F**) wound healing assay analysis (**E**) with representative images (**F**). *: *p* < 0.05, **: *p* < 0.01, ***: *p* < 0.001; one-way ANOVA followed by post hoc analysis; #: *p* = 0.051 using one-way ANOVA followed by Tukey’s post hoc analysis and *p* < 0.05 using one-way ANOVA followed by pairwise analysis.

We evaluated and confirmed the deregulation of the miR-506 in the respective cell groups using qPCR. As shown in Figure 3A, the sorted cells that survived the lentiviral transduction and antibiotic treatment presented a respective upregulated or downregulated miR-506 expression. Briefly, cells in the miR-506 upregulation group demonstrated a >154-fold increase in the miR-506 levels compared to the miR-control group (*p* < 0.001), while the cells in the miR-506 downregulation group demonstrated a 92% decrease in miR-506 levels compared to the miR-control group (*p* < 0.001).

#### 2.3.2. miR-506 Downregulation, but Not Upregulation, Affected the Cell Cycle, but Both Slowed Down Proliferation

Briefly, miR-506 upregulation did not affect the cell cycle population distribution, presenting no significant differences compared to the miR-control group for any of the cell cycle phases. In contrast, miR-506 downregulation altered the cell cycle population distributions, as further analyzed below. Interestingly, both the miR-506 upregulation and downregulation groups demonstrated an increased cell doubling time.

In more detail, the miR-506 upregulation induced (Figure 3B) a 4% decrease in the G0/G1 cell population compared to the miR-GFP control (no significance), while the miR-506 downregulation was lower than the miR-control by ~7% (*p* = 0.051 for ANOVA and Tukey’s post hoc analysis and *p* < 0.05 for ANOVA followed by pairwise comparison). This suggests that miR-506 downregulation decreased the G0/G1 cell population by ~3% more than the miR-506 upregulation, even though the population distribution was lower than in the miR-control group in both cases. For the S population, the miR-506 upregulation increased its cell population by 3% compared to both the miR-control group and miR-506, though there were no statistically significant differences among the groups. Similarly, miR-506 upregulation made no significant difference in the G2 cell population compared to the miR-control group. Interestingly, the miR-506 downregulation had a significantly higher G2 population, having a ~1.9-fold and ~2-fold higher G2 population compared to the miR-506 upregulation and miR-control groups, respectively (*p* < 0.01 for both).

Furthermore, the cell doubling time analysis (Figure 3C) indicated that both the miR-506 upregulation and downregulation increased the cell doubling time for the cells, with miR-506 upregulation significantly increasing the cell doubling time by 5.6% (*p* < 0.001) and the miR-506 downregulation increasing the cell doubling time by 5.1% (no significance) compared to the miR-control group. Furthermore, there was no significant difference between the miR-506 upregulation and the miR-506 downregulation.

#### 2.3.3. The Two miR-506 Deregulations Altered Cell Morphology in a Similar Manner, but Not Motility

Through QPI (Figure 3D), we identified how miR-506 deregulation affected three morphometric characteristics in the A549 cells. Briefly, both miR-506 upregulation and downregulation induced similar changes to the cells’ sphericity, perimeter, and area. For the sphericity, both miR-506 upregulation and downregulation decreased the cells’ sphericity, with the former representing a 12.4% decrease and the latter representing a 6% decrease in sphericity compared to the miR-control group. All differences were statistically significant among the groups. In contrast, the cellular perimeter for miR-506 upregulation was increased by 19.2% compared to miR-control (*p* < 0.001), while the miR-506 downregulation was increased by 7.9% (*p* < 0.001). This would account for miR-506 upregulation inducing a 10.5% larger perimeter than miR-506 downregulation (*p* < 0.001), despite both groups having higher values than the control. Similarly, the cell area for the miR-506 upregulation and downregulation groups was increased by 35% and 12.1%, respectively, compared to the miR-control group (*p* < 0.001 for both). This accounts for a 20% larger surface area induced by miR-506 upregulation compared to the respective miR-506 downregulation.

Based on the wound healing assay (Figure 3E,F), miR-506 upregulation overall slightly reduced the rate of closure compared to the miR-control for the 24 and 48 h time points, where the miR-control presented a 1.22-fold and 1.16-fold larger closed area compared to the miR-506 upregulation for the two time points, respectively. In both cases, though, these differences were not statistically significant. In contrast, miR-506 downregulation presented larger area closure compared to both the miR-upregulation and miR-control groups. In more detail, the miR-506 downregulation cells presented a 2.12-fold larger closed area compared to the miR-506 upregulation cells (*p* < 0.001) and 1.74-fold larger closed area compared to miR-control (*p* < 0.001) at 24 h, while for the 48 h time point, the miR-506 downregulation cells presented a 1.41-fold larger closed area compared to miR-506 upregulation (*p* < 0.05) and 1.22-fold larger closed area compared to the miR-control group (*p* < 0.05).

### 2.4. miR-506 and miR-143 Deregulation

#### 2.4.1. Dual Lentiviral Transduction Induced Both miR-506 and miR-143 Deregulation

**Figure 4:**
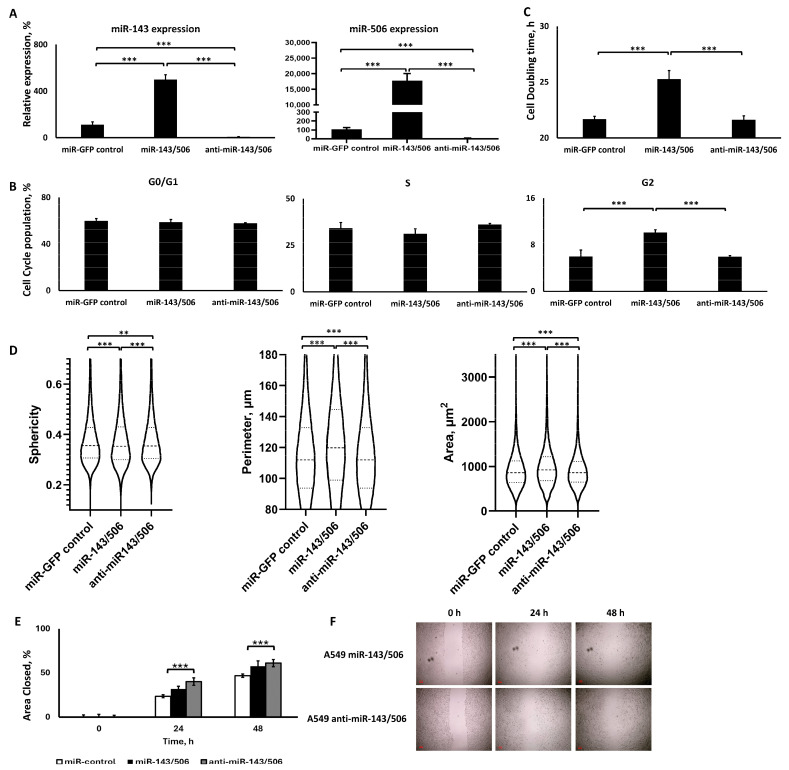
Analysis of the A549 cells transduced using lentiviruses for dual miR-143/506 up- or downregulation. (**A**) qPCR analysis of miR-143 (left) and miR-506 (right) expressions; (**B**) cell cycle analysis; (**C**) cell doubling time analysis; (**D**) QPI morphometric analyses for sphericity, perimeter, and area of the cells over a 24 h period; (**E**,**F**) wound healing assay analysis (**E**) and representative images (**F**). **: *p* < 0.01, ***: *p* < 0.001; one-way ANOVA followed by post hoc analysis.

We evaluated and confirmed the deregulation of miR-506 and miR-143 in the respective groups. As shown in Figure 4A, qPCR analysis on the sorted cells that survived the lentiviral transduction and antibiotic treatment indicated that the lentiviral transduction upregulated or downregulated both miR-506 and miR-143 expressions for the respective cell groups. In more detail, our approach first included the stable transduction using lentiviruses for miR-506 up- or downregulation in A549 cells. Following transduction, G418 treatment for selection (850 μg/mL) for up to 14 days, and qPCR confirmation for miR-506 upregulation or downregulation in the respective cell groups, the cells were treated with lentiviruses for the upregulation or downregulation of miR-143 to the respective upregulated and downregulated miR-506 cells. Following lentiviral transduction, the cells were treated with puromycin (2.5 μg/mL), and qPCR analysis took place to confirm the dual miR up- or downregulation. Subsequently, the cells were sorted and were introduced to a cyclic antibiotic treatment of puromycin followed by G418 to maintain the transductions. Cells in the miR-143/506 upregulation group demonstrated a >5-fold increase in miR-143 expression and >177-fold increase in miR-506 expression compared to the miR-control group (*p* < 0.001), while the cells in the miR-143/506 downregulation group demonstrated a 92% decrease in miR-143 expression and 91% decrease in miR-506 expression compared to the miR-control group (*p* < 0.001). These confirm that the dual up- or downregulations were successful.

#### 2.4.2. miR-143/506 Upregulation Increased G2 Cell Population and Slowed Down Proliferation

Briefly, miR-143/506 upregulation significantly increased the G2 population in the cell cycle cell distribution and the cell doubling time compared to miR-control and miR-143/506 downregulation. In contrast, the miR-143/506 downregulation did not present any significant differences compared to miR-control for either cell cycle phase cell distribution or cell doubling time. In more detail, miR-143/506 upregulation did not present any appreciable differences in the G1 and S cell populations compared to miR-control or miR-143/506 downregulation (Figure 4B), although the S population was slightly lower for the miR-143/506 upregulation group compared to miR-control (~3% lower) and miR-143/506 downregulation (~5% lower), without achieving statistical significance. In contrast, miR-143/506 upregulation induced a significant 1.7-fold increase in the G2 cell population compared to both miR-control (*p* < 0.001) and miR-143/506 downregulation (*p* < 0.001).

Furthermore, miR-143/506 upregulation increased the cell doubling time (Figure 4C) for the cells compared to miR-control and miR-143/506 downregulation, with miR-143/506 upregulation significantly increasing the cell doubling time by 5.4% (*p* < 0.001) compared to miR-control and by 4.6% (*p* < 0.001) compared to miR-143/506 downregulation. There was no significant difference between miR-143/506 downregulation and miR-control.

#### 2.4.3. miR-143/506 Upregulation Altered Cell Morphology, but miR-143/506 Downregulation Increased Cell Motility 

Through QPI, we identified that miR-143/506 upregulation altered certain morphometric characteristics in the A549 cells, while the cells in the miR-143/506 downregulation group did not present any appreciable differences from the cells in the miR-control group (Figure 4D). In more detail, miR-143/506 deregulation did not appreciably alter the sphericity of the cells, producing a ~1% increase for the dual miR upregulation and 0.46% decrease for the dual miR downregulation in sphericity compared to miR-control, though in both cases these small differences were statistically different. In contrast, miR-143/506 upregulation increased the cells’ area by 10.2% (*p* < 0.001) and the perimeter by 8.8% (*p* < 0.001) compared to miR-control, while miR-143/506 downregulation had a significantly smaller effect on these two parameters, producing a 1.8% (*p* < 0.001) increase in area and 0.93% (*p* < 0.001) increase in perimeter compared to miR-control. Thus, the miR-143/506 downregulation cells presented significantly lower values for both area and perimeter.

Based on the wound healing assay, miR-143/506 upregulation overall did not present considerable differences compared to miR-control, presenting only a 1.35-fold and 1.23-fold larger area closed for the 24 and 48 h time points, respectively, with these differences not being statistically significant. The cells with miR-143/506 downregulation closed a 1.71-fold larger area (*p* < 0.001) for the 24 h time point compared to miR-control, while for the 48 h time point, the closed area difference between the two groups was reduced, with the miR-143/506 downregulation being 1.3-fold higher than the miR-control (*p* < 0.01). Interestingly, the miR-143/506 downregulation had an accelerated wound gap closure rate at both time points compared to miR-143/506 upregulation, though there was no statistically significant difference between the two groups for either time point.

## 3. Discussion

Lung cancer is the primary cause of cancer-related deaths in the United States [1]. Novel therapeutic approaches are necessary to combat this disease. miRs have emerged as essential regulators in multiple diseases, and identifying their function is an on-going, extensive, and complicated procedure. This is also evident in the extensive literature currently available on miR studies. Traditionally, transient transfections of miRs to cells have been a reliable approach for their evaluation in different cells/diseases. Such approaches have been the cornerstone of miR evaluations as they allow the simple assessment of an acute treatment in vitro or in vivo, which resembles a therapeutic evaluation in many ways. 

Alternatively, stable up-/downregulation of miRs of interest through lentiviral transduction can also be utilized to evaluate the cell responses over prolonged miR deregulation [12,34,35,36]. Although this may not resemble a therapeutic application, the approach can elucidate cellular mechanistic activities due to the induced miR deregulation, which can frequently correlate to an acute treatment. On the other hand, if the studied acute miR deregulations induce apoptosis, stable deregulations may be unable to resemble cellular behavior under an acute transfection, as the surviving cells may represent cells that have evaded the apoptotic pressure. However, evaluating the cells that survive the stable miR deregulation’s apoptotic stress can demonstrate how the cells would behave after prolonged acute miR treatments, comparable to evaluating a cell developing resistance to the treatment. This vital information would indicate whether a miR deregulation can potentially present any unexpected or undesired effects after prolonged treatment. 

Our previous work [9,18,33] evaluated how miR-143 and miR-506 affect the cell cycle progression in LC cells, with our work expanding to pancreatic cancer cells. The combinatorial miR indicated a cell cycle arrest, which was attributed to the effect of miR-506 on CDK4/6 expressions [24,25] and miR-143 on CDK1 expression [26]. Cell cycle arrest is associated with preventing cell cycle progression and eventual cell apoptosis [37]. Furthermore, the miR-143/506 combinatorial treatment indicated strong apoptotic activity in the cancer cells. The effects of these two miRs individually were also reported in prior or later publications to our work by additional groups in various cancers, including lung cancer (representative literature: miR-506: [12,24,31]; miR-143: [26,27,32]).

In the present study, we evaluated the effects of stable deregulation of miR-143 and/or miR-506. Although our interest lies predominantly in the miR combination, we assessed the individual miR deregulations to understand the combinatorial effect better and ensure a comprehensive understanding.

miR-143 and miR-506 were previously reported to have significant downregulation in LC cells compared to normal cells, as we [9] and others [30,31] previously reported. However, it is necessary to point out that miR-143’s downregulation in LC cells was more pronounced and ubiquitous than miR-506’s. We performed a lentiviral transduction, which induced a stable up- or downregulation for the respective miRs. The dual miR deregulation induced comparable changes in the respective miR expression compared to the respective individual miR deregulations, as detected via qPCR. As single and dual miR upregulations have been reported to induce apoptosis, it is reasonable to conclude that the cells that survived the miR deregulation are those that had the capacity to evade apoptosis. 

We first evaluated the effects of miR deregulation on the cell cycle. The miR-143 upregulation alone elicited a modest increase in the G1 cell population and a significant decrease in the S population compared to the miR-control and miR-143 downregulation groups. More importantly, the miR-143 upregulation increased the G2 cell population considerably. Interestingly, the G2 cell population for the miR-143 downregulation was also elevated, demonstrating a comparable result to the respective upregulation, although this effect did not achieve statistical significance. These results aligned with our previous work on A549 cells and their transient transfection using miR-143 mimics, where we observed an increase in the G1 cell population, a decrease in the S cell population, and a slight increase in the G2 population, though our analysis did not include the evaluation of a miR-143 inhibitor [18].

We evaluated the cell doubling time using QPI analysis with Livecyte to determine whether these cell cycle population changes due to the miR-143 deregulations translate to changes in cell proliferation. Our analysis did not indicate any changes in the cell doubling time for either miR-143 up- or downregulation, although miR-143 upregulation indicated significantly decreased S and increased G2 phase cell populations. In fact, the miR-143 upregulation group indicated a slightly decreased cell doubling time, though it was not a statistically significant difference. It would have been anticipated that miR-143 upregulation would present the opposite effect on the cell cycle compared to the respective miR-143 downregulation. However, although only the miR-143 upregulation significantly decreased the S population, the miR-143 downregulation also presented an elevated G2 population, albeit not a significant one vs. miR-control or miR-143 upregulation. Similarly, the miR-506 stable upregulation did not induce any significant alterations to the cell cycle, although our prior work indicated an increase in the G1 cell population using miR-506 mimic transient transfections [18].

These unexpected behaviors may be attributed to the fact that miR-506, as well as miR-143, have been reported to induce apoptotic activity in A549 cells [8,30], and the results we observe originate from the cells capable of evading the treatment’s apoptotic pressure. This is also supported by previous work with CDK inhibitors in lung [38,39] or breast cancer cells [40,41,42], where cells with developed resistance to CDK inhibitors present a limited response to these compounds, with minimal inhibition of the cell cycle progression and elevated CDK4 or 6 expressions. Interestingly, stable miR-506 upregulation in pancreatic cancer cells was reported to induce a G1 cell population increase, which may indicate a cancer-specific outcome [12]. In contrast, miR-506 downregulation significantly decreased the G1 cell population compared to the miR-control group but not to the miR-506 upregulation group, while significantly increasing the G2 cell population compared to both miR-control and miR-506 upregulation. Evaluating the cell proliferation rate, miR-506 upregulation significantly increased the cell doubling time for the cells compared to miR-control but not to miR-506 downregulation. Thus, similarly to the miR-143 deregulations, two opposite deregulations did not present the opposite effect, which could be attributed to the cells developing a compensatory mechanism for the miR’s effects. Nonetheless, it is evident that the single miR deregulations do not produce a well-defined or miR-expression-dependent effect. 

Our previous analysis in LC cells [9,18,33] indicated a G1/S and G2/M transition inhibition when transiently transfecting both miR-143 and miR-506 mimics for 24 and 48 h. Here, stable upregulation of both miRs in A549 cells did not alter the cell population in the G1 phase, though they caused a slight decrease in the S phase cell population. This result of the dual upregulation would appear to resemble the single miR-143 upregulation above, though the effect was not significant for the dual upregulation. In contrast, the G2 phase cell population for the dual miR upregulation was significantly increased compared to both the miR-control and miR-143/506 downregulation, for which the latter did not demonstrate any appreciable difference from the control group. This is in contrast to the individual miR deregulations, which did not present a well-defined response that correlated to the levels of the miR. Furthermore, the dual miR upregulation induced a significant increase in the cell doubling time compared to the control and the respective downregulation, which also indicates a well-defined effect that depends on miR expression, in contrast to the individual miR deregulations. As we saw, both miR-506 deregulations increased the doubling time, while neither of the miR-143 deregulations significantly altered the cell’s doubling time compared to the control group. 

Subsequently, we utilized Livecyte quantitative phase imaging to identify changes in the morphometric characteristics of the cells due to miR deregulation. The morphometric analysis of the cells with the various miR deregulations further supported the opinion that the individual miR deregulations did not present an identifiable effect that correlated to the miR levels, while the dual miR deregulation did. Briefly, the miR-143/506 upregulation demonstrated higher sphericity, perimeter, and area. These differences were present compared to either the control or dual miR-downregulation groups. Furthermore, these results are in contrast to miR-506 deregulation, in which both up- and downregulations decreased sphericity while increasing area and perimeter; and both miR-143 deregulations decreased sphericity, while only miR-143 downregulation increased area and perimeter.

Finally, we performed wound healing assays to evaluate changes in cell motility due to the miR deregulations. Our analysis indicated that only the miR-506 upregulation (at least compared to miR-506 downregulation) demonstrated significantly decreased area closure. This finding aligns with the prior literature, in which miR-506 upregulation was associated with decreased migration in various cancers [7,43,44]). In a similar manner, the miR-143 downregulation also presented an increased area closure compared to both the control and the respective individual upregulation group at the 24 h and 48 h time points. We need to point out that there have been conflicting reports on miR-143 upregulation, in which it has been stated to either increase or decrease the migration of LC cells [35,45,46,47,48,49]. However, the miR-143/506 upregulation indicated no appreciable differences in area closure compared to miR-control. More importantly, the miR-143/506 downregulation presented an increase in the area closure, in contrast to miR-143/506 upregulation and in alignment with the two individual miR downregulations. It is worth noting that the data presented on the A549 non-small cell lung cancer cell line correspond to a lung adenocarcinoma cell line, which specifically carries KRAS and CDKN2A gene mutation, with KRAS being one of the most commonly observed mutations in LC [50]. Several cell lines and animal models have been developed over the years to evaluate different aspects of the disease [51]. With lung adenocarcinoma having the highest frequency among all types of LCs, A549 is regarded as a good representative cell line for the disease and is frequently and predominantly studied. Nonetheless, it is important to evaluate the effect of miR-143/506 stable upregulation on additional NSCLCs with other driver mutations.

From our analysis, it is reasonable to conclude that although the individual miRs can have a beneficial effect on LC cells short-term, long-term exposure may not present the same benefits as the acute treatment. This is also supported by the fact that differences in several analyses between respective individual miR upregulation vs. downregulation become unclear or similar, or the observed effect does not appear to depend on the levels of the miRs. In contrast, dual miR upregulation appears to not only be beneficial during short/acute treatments; even under prolonged treatments, the benefits would primarily be maintained. Furthermore, the dual miR upregulation maintained an apparent beneficial, miR-expression-dependent effect, while the dual miR downregulation did not possess these characteristics. As the standard of care for cancer patients today frequently involves drug combinations [52], although consideration on potential side effects needs to take place, our findings support our initial approach that a dual (or more) miR regulation presents significant advantages compared to a single miR regulation [18], and the particular miR combination merits further evaluation.

## 4. Materials and Methods

### 4.1. Materials

The A549 human lung adenocarcinoma cell line (cat no. CCL-185), Universal Mycoplasma Detection Kit (cat no. 30-1012K), and FTA Sample Collection Kit for Human Cell Authentication Service (cat no. 135-XV) were purchased from ATCC (Manassas, VA, USA). All lentiviruses were obtained from Applied Biological Materials (cat nos. m002, mh15183, mh35190, mh16154, mh35891; vector maps are presented in Supplementary Figure S1) (Richmond, BC, Canada). Hexadimethrine bromide (cat no. H9268) was purchased from Millipore, Sigma (St. Louis, MO, USA). TaqMan qPCR microRNA primers (hsa-miR-143-5x, -20x; hsa-miR-506-5x, -20x; and U6 housekeeping gene-5x, -20x) were purchased from Thermo Fisher Scientific (cat nos. 4427975(002249), 4427975(001050), and 4427975(001973)) (Waltham, MA, USA). Cell culture medium DMEM was purchased from VWR (Radnor, PA, USA), and F12K media were purchased from Corning (Corning, NY, USA). Penicillin/streptomycin was purchased from VWR. Puromycin (CAS #53-79-2) and Invivogen G418 (cat no. NC9107150) were purchased from Fisher Scientific (Waltham, MA, USA). Fetal bovine serum was obtained from Biotechne (Minneapolis, MN, USA). A 1kb DNA step ladder was obtained from Promega (Madison, WI, USA).

### 4.2. Cell Culture

A549 parent cells and A549 stably transduced cells were cultured in DMEM/F-12K (1:1) complete medium supplemented with 10% fetal bovine serum (FBS) and 1% penicillin/streptomycin. Cells were cultured at 37 °C, under a 5% CO_2_ humidified atmosphere. The culture media for A549-miR-GFP control, -miR-143, and -anti-miR-143 were additionally supplemented with 2.5 µg/mL puromycin, and A549-miR-506 and A549-anti-miR-506 were supplemented with 850 µg/mL G418. Additionally, for the combination of stably transduced groups, i.e., A549 miR-143/506 and anti-miR-143/506, an initial 10–14-day treatment with 850 µg/mL G418 was followed by 3-5 days of 2.5 µg/mL puromycin treatment, with the described antibiotic treatment cycle subsequently repeating. 

### 4.3. Stable Cell Line Generation

A549 cells were seeded in 24-well plates at a seeding density of 5 × 10^4^ cells/well overnight. The following day, the culture media in the wells were replaced with complete media containing 8 µg/mL hexadimethrine bromide. A calculated volume of viral particles (viral titer > 10^7^ IU/mL) was added at a multiplicity of infection (MOI) of 5. The cells were then incubated with the viral particles for 24 h. The following day, the media in the wells were replaced by fresh complete media, followed by splitting the cells in 1:3 and seeding them in a new 24-well plate for a 48 h incubation. We determined the respective concentrations of the selection antibiotic by performing a kill curve experiment. Briefly, we seeded A549 parent cells in a 24-well plate with varying concentrations of puromycin (0.5–5 µg/mL) for 5 days and G418 (100–1000 µg/mL) for 10 days in duplicates. Based on cell survival at different concentrations, we selected the appropriate concentration, which for puromycin was 2.5 µg/mL and for G418 was 850 µg/mL. These concentrations were used to treat the newly transduced cells for the appropriate antibiotic for selection. miR control-GFP viral transduction in A549 cells was used as a negative control. 

### 4.4. Cell Sorting

All stably transduced cells were sorted using a BD FACS Aria III Cell Sorter (BD Biosciences, Franklin Lakes, NJ, USA). Initially, we harvested 5–8 × 10^6^ A549 stably transduced cells from each group and washed them with 1× sterile phosphate-buffered saline (PBS). We prepared cell suspensions in 2 mL sterile PBS (1×) via gently mixing and filtering through a 40-micron filter. We fixed the forward scatter (FSC) versus the side-scatter (SSC) gating using untagged A549 cells. Subsequently, we gated the cell population with positive GFP fluorescence, which we used to sort and collect the cells in a sterile 15 mL falcon tube containing 3 mL complete media. The sorted cells were subsequently used for RNA extraction and seeding, followed by the respective antibiotic treatment for selection after one day in culture. 

### 4.5. RNA Extraction and qPCR Analysis

We conducted qPCR analysis as previously described [18,33,53]. In brief, we extracted total RNA from cell samples in triplicates using the Quick-RNA miniprep kit (Zymo Research, Irvine, CA, USA) and following the manufacturer’s protocol. We quantified RNA concentration using Nanodrop, followed by miR cDNA synthesis using TaqMan™ MicroRNA Reverse Transcription Kit (Thermo Fisher, Waltham, MA, USA) following the manufacturer’s protocol. We conducted RT-qPCR to detect miRNA expression for hsa-miR-143-3p and hsa-miR-506-3p, with U6 snRNA serving as the housekeeping gene, using a BioRad CFX96 real-time PCR system (BioRad systems, Hercules, CA, USA) and TaqMan Universal Master Mix II assay along with the respective TaqMan miRNA specific primers for miRNA expression (Applied Biosystem, Carlsbad, CA, USA). Relative miRNA expression was calculated using ∆∆Cq analysis. 

### 4.6. Cell Cycle Analysis

We performed cell cycle analysis as previously described [18]. First, we harvested each A549 stably transduced cell group individually. Following PBS washing, the cells were fixed in 3.7% paraformaldehyde and fixed in 70% ethanol. Subsequently, we stained the cells with propidium iodide (MP Biomedicals, LLC, Santa Ana, CA, USA) and ribonuclease-A (Sigma), and analyzed them using a BD FACS Calibur Flow Cytometer, with Cellquest Pro v. 6.0 software (BD Biosciences, Franklin Lakes, NJ, USA). We processed the data we obtained using ModFit LT 5.0 (Verity Software House, Topsham, ME, USA; Supplementary Figure S5). The cell cycle data are presented as the percentage of cell distributions in the different phases (G0/G1, S, and G2). 

### 4.7. Wound Healing Assay

We seeded 0.25 × 10^6^ cells/well in a 24-well plate in 3 wells, as replicates, and incubated the cells overnight in the conditions described above. After the cells reached 95–100% confluency, we gently scratched the middle of the well along the diameter of the well using a 200 µL pipette tip. We gently washed the cells 3 times with PBS to remove debris and added 2% FBS-containing media. We obtained images of the formed gap under the microscope at 0, 24, and 48 h [33]. We processed all the images using Image J 1.53k software. The data are presented as the percentage area of the wound closed at the pre-determined time points (imagej.nih.gov; last accessed: 15 March 2024).

### 4.8. Mycoplasma Detection Analysis

We performed mycoplasma detection analysis following the manufacturer’s protocol. Briefly, cells were harvested and lysed, and we performed PCR using primers provided by the vendor (Universal Mycoplasma Detection Kit; cat no. 30-1012K). Bands were detected using ethidium bromide-stained gel electrophoresis.

### 4.9. Quantitative Phase Imaging

We seeded 2 × 10^4^ A549 stably transduced cells/well overnight in 24-well glass bottom plates (24-well glass bottom plates, Cellvis cat no. P24-1.5H-N) in 3 wells per group. The next day, cells were rinsed twice with sterile PBS to remove any dead cells or debris, and a fresh complete medium was added to each well. The plate was then carefully mounted onto the Livecyte Kinetic Cytometer (Phasefocus, Sheffield, UK) and constant acquisition was commenced at pre-determined time points for 24 h. We quantified the cell doubling time, area, perimeter, and sphericity [54]. 

### 4.10. Statistical Analyses

We analyzed the obtained data using a one-way analysis of variance (ANOVA) followed by a post hoc Tukey’s test, unless stated otherwise, to determine the significance of differences among the groups. All statistical analyses were performed using at least triplicates per experiment unless stated otherwise. We have presented all data as mean data ± standard error (SE) of the mean. *p*-values ≤ 0.05 are considered statistically significant.

## 5. Conclusions

This study analyzed the effect of dual stable miR-143/506 upregulation on LC cells to evaluate the effects of prolonged and sustained miR exposure. The cells that survived the transduction and the acute apoptotic activity of the dual miR-143/506 upregulation maintained a behavior that could prove advantageous for regulating cancer progression, comparable to the acute treatment. This effect was miR-expression-dependent, thus opposite of the respective miR-143/506 downregulation, and included increased cell doubling time and increased G2 cell cycle population. These results were in contrast to the individual miR-143 or miR-506 deregulations, of which the respective up- or downregulation did not indicate an opposing outcome dependent on the opposing miR levels. This was observed in our measurements of their respective cell doubling time, the cell cycle analysis, or the cells’ morphometric characteristics. Thus, our analysis supports that the proposed miR combination can be beneficial against LC cells, unlike the individual miRs alone, and even in the scenario of a potential prolonged miR-143/506 treatment. Consequently, the proposed treatment maintains significant advantages compared to the control group, miR-143/506 downregulation, or the individual miR deregulations, which merits further evaluation. 

## Data Availability

The data presented in this study are available on request.

## Supplementary Materials

The following supporting information can be downloaded at https://www.mdpi.com/article/10.3390/ijms25084432/s1.
