# Supplementary material for: Stable Dual miR-143 and miR-506 Upregulation Inhibits Proliferation and Cell Cycle Progression
Source: Int J Mol Sci. 2024 Apr 17;25(8):4432. doi: 10.3390/ijms25084432 (PMC11050449; doi:10.3390/ijms25084432)

Stable Dual miR-143 and miR-506 Upregulation Inhibits Proliferation and Cell Cycle Progression

Archana Shrestha[1], Behnaz Lahooti[2], AKM Nawshad Hossian[1], Mahboubeh Madadi[3], Constantinos M. Mikelis[2, 4], George Mattheolabakis[1, *]

Supplementary Figure S1: Vector maps for lentiviruses used to induce miRNA deregulation to the A549 cells. Vector maps were provided by the vendor (ABMgood).

LentimiRa-GFP-hsa-miR-143-3p Virus

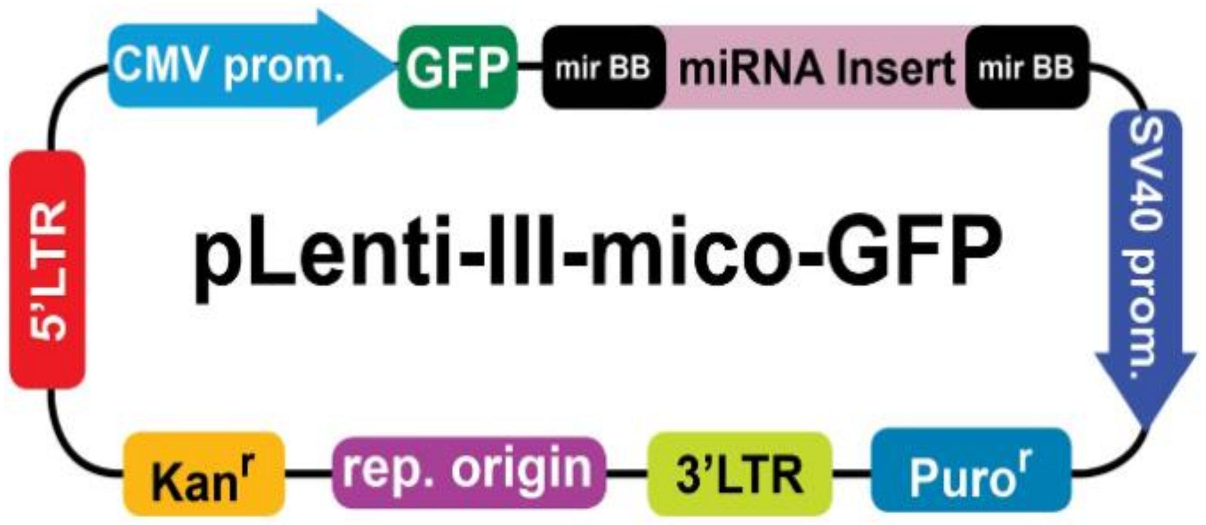

Lenti-miRa-GFP-hsa-miR-506-3p Virus (Neo)

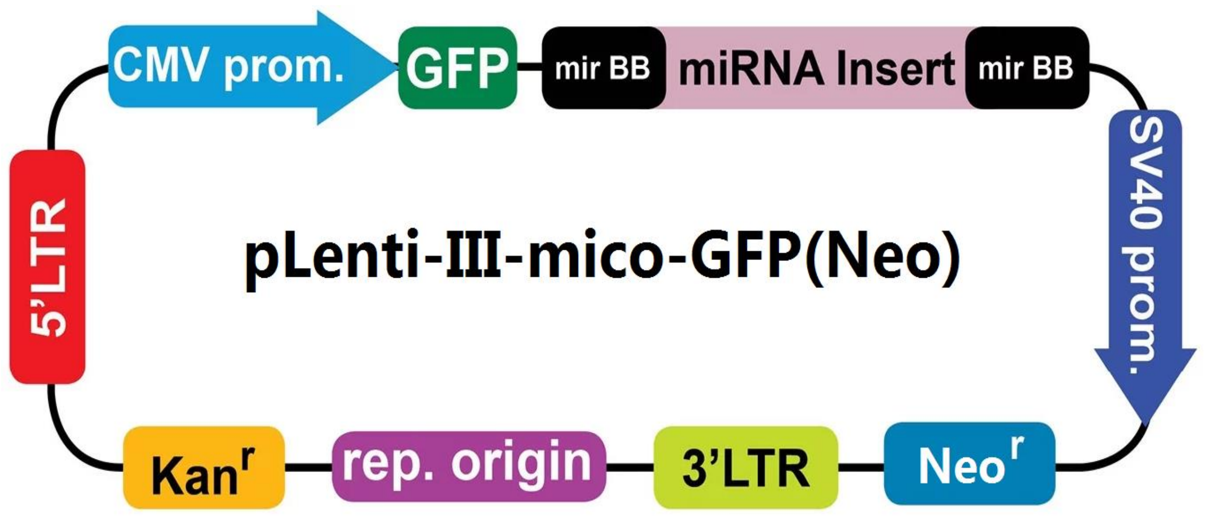

LentimiRa-Off-hsa-miR-143-3p Virus

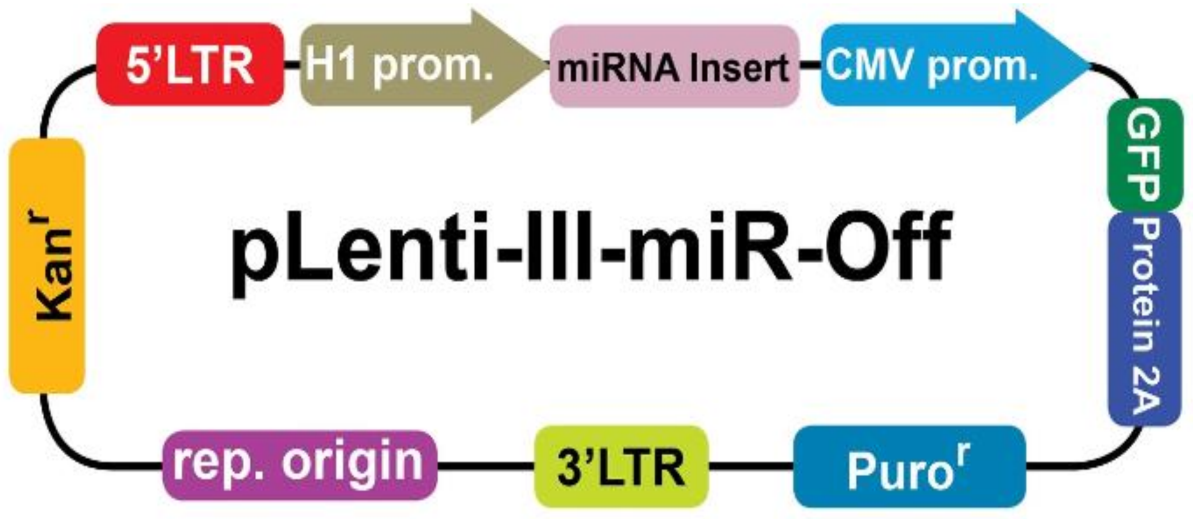

Lenti-miRa-Off-hsa-miR-506-3p Virus (Neo)

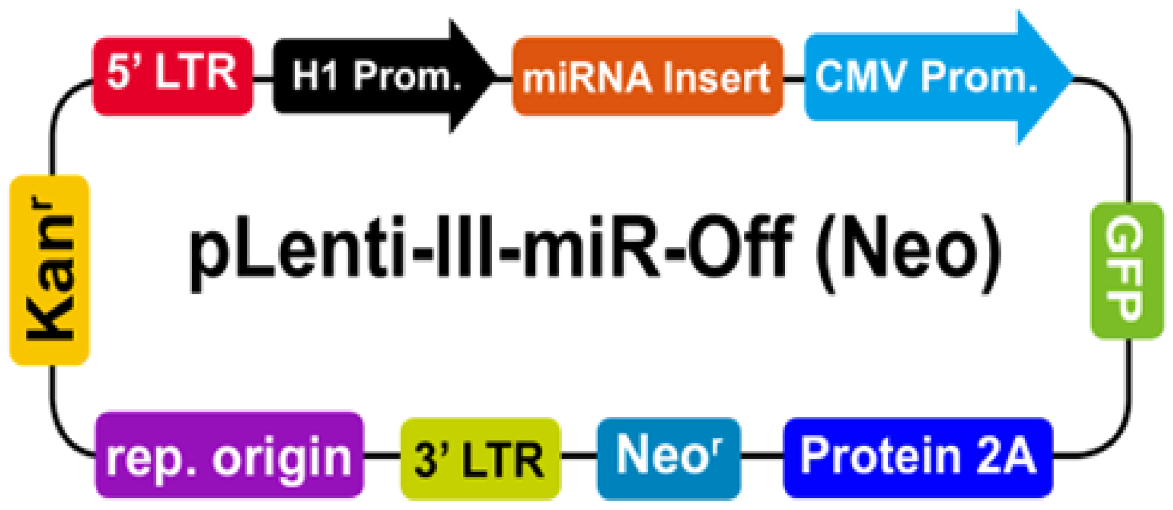

Lenti-III-mir-GFP Control Virus

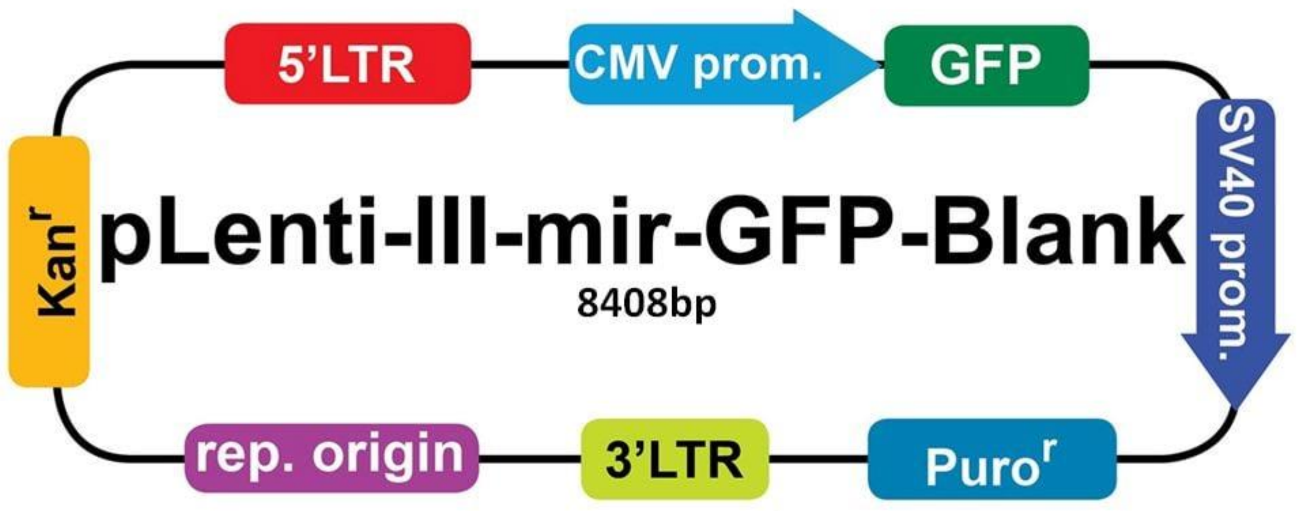

Supplementary Figure S2: Cell sorting of A549 cells transduced using lentiviral particles for miR deregulation. GFP signal was used for selecting cells.

Sorting analysis of A549-miR-control cells

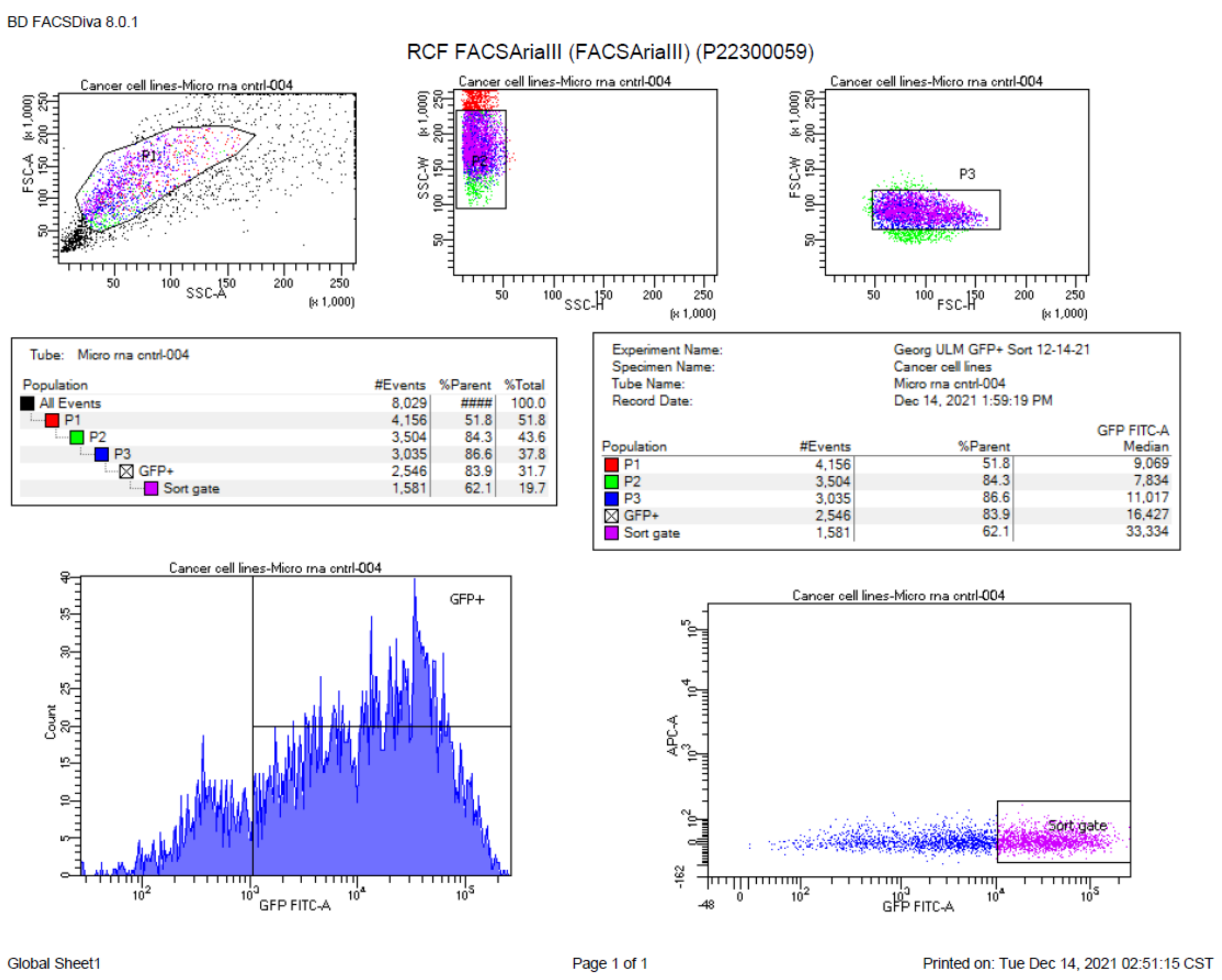

Sorting analysis of A549-miR-143 cells

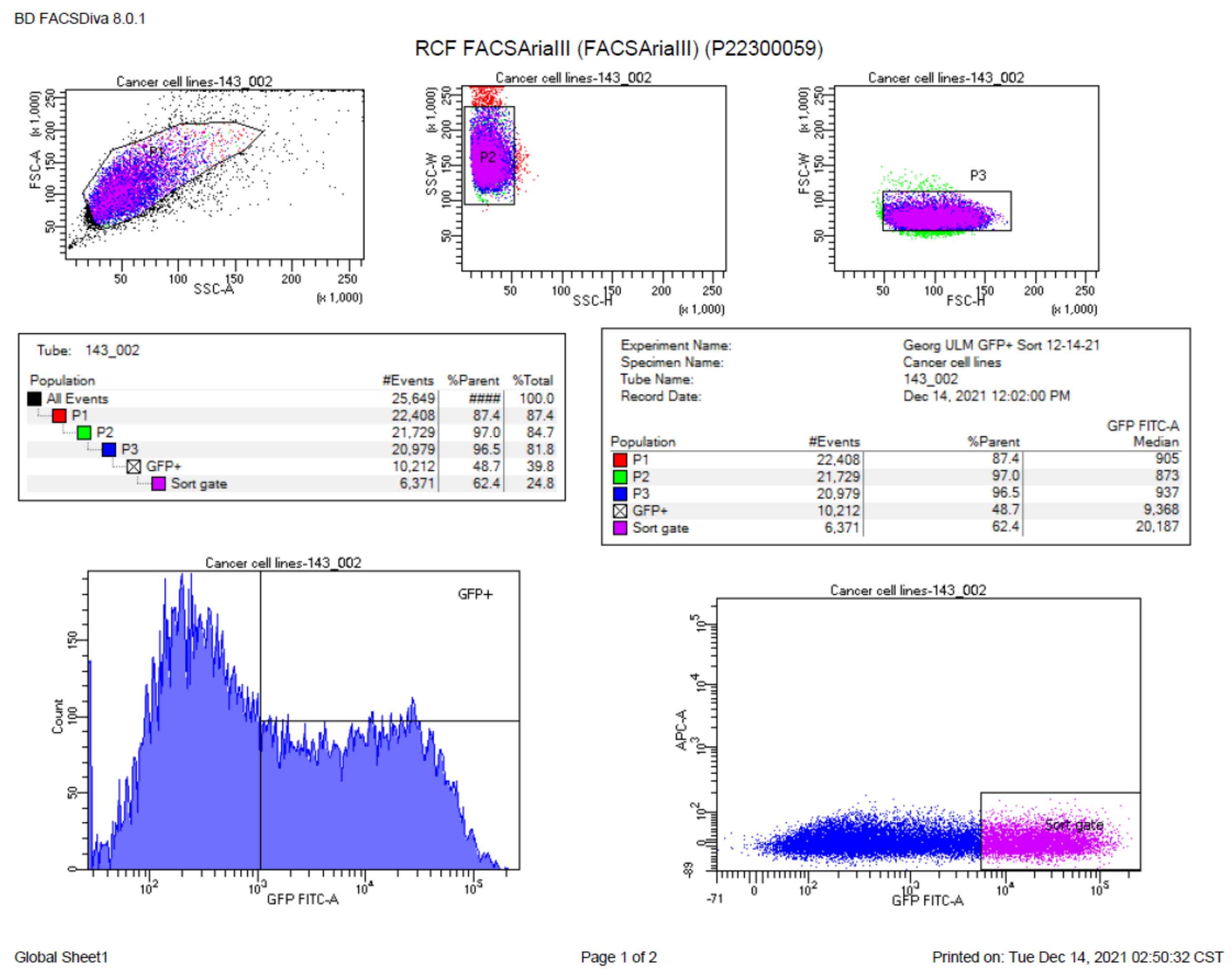

Sorting analysis of A549-anti-miR-143 cells

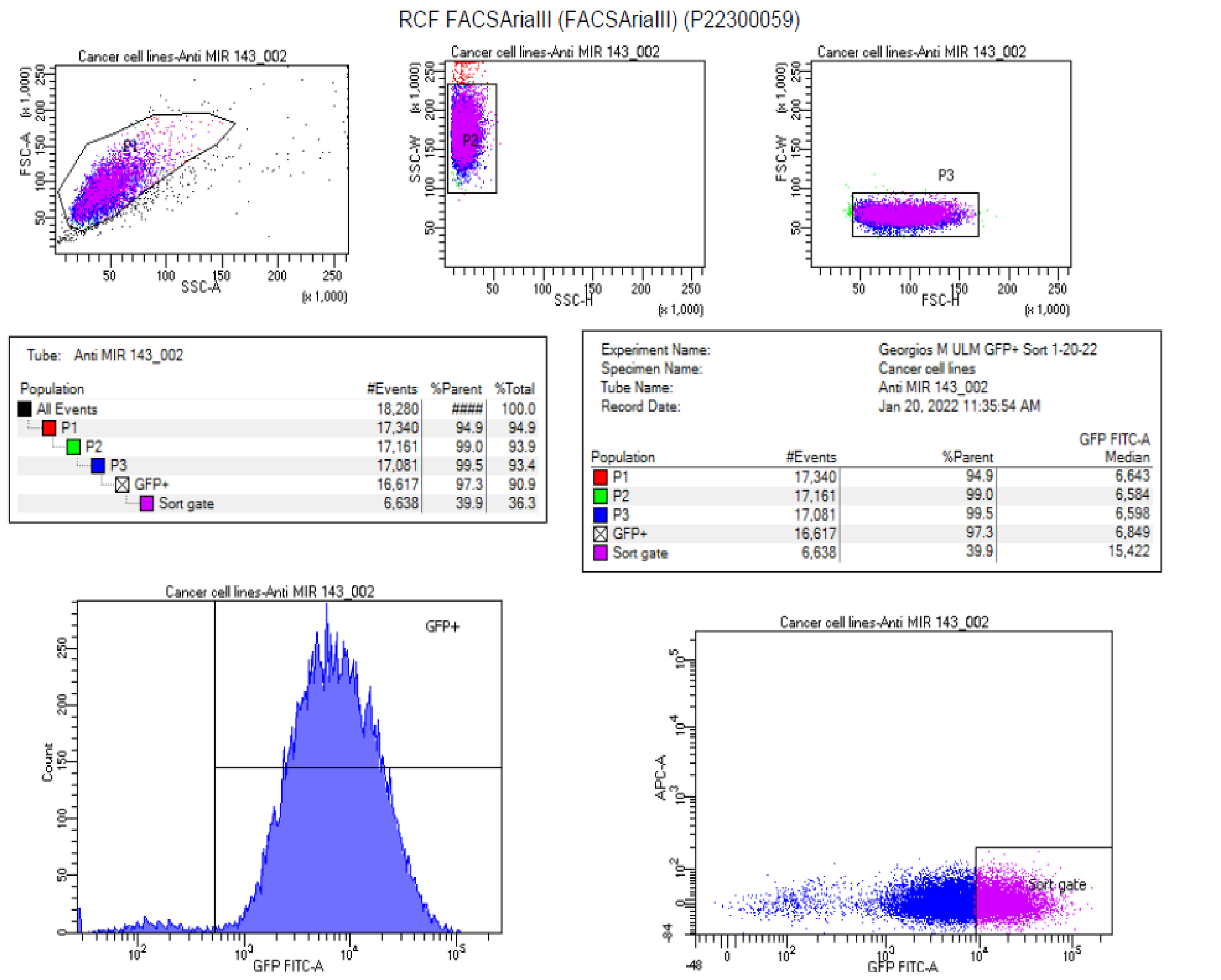

Sorting analysis of A549-miR-506 cells

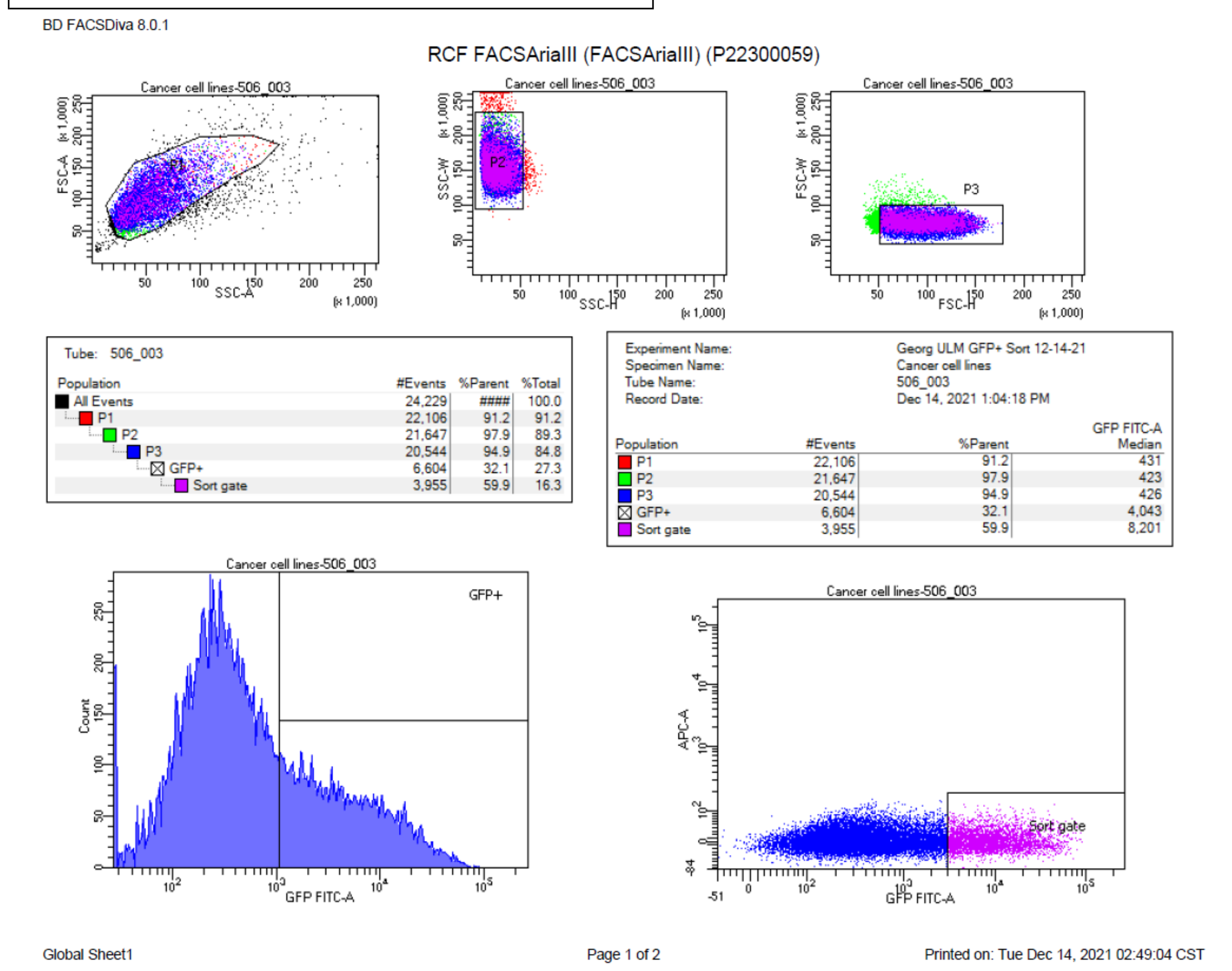

Sorting analysis of A549-anti-miR-506 cells

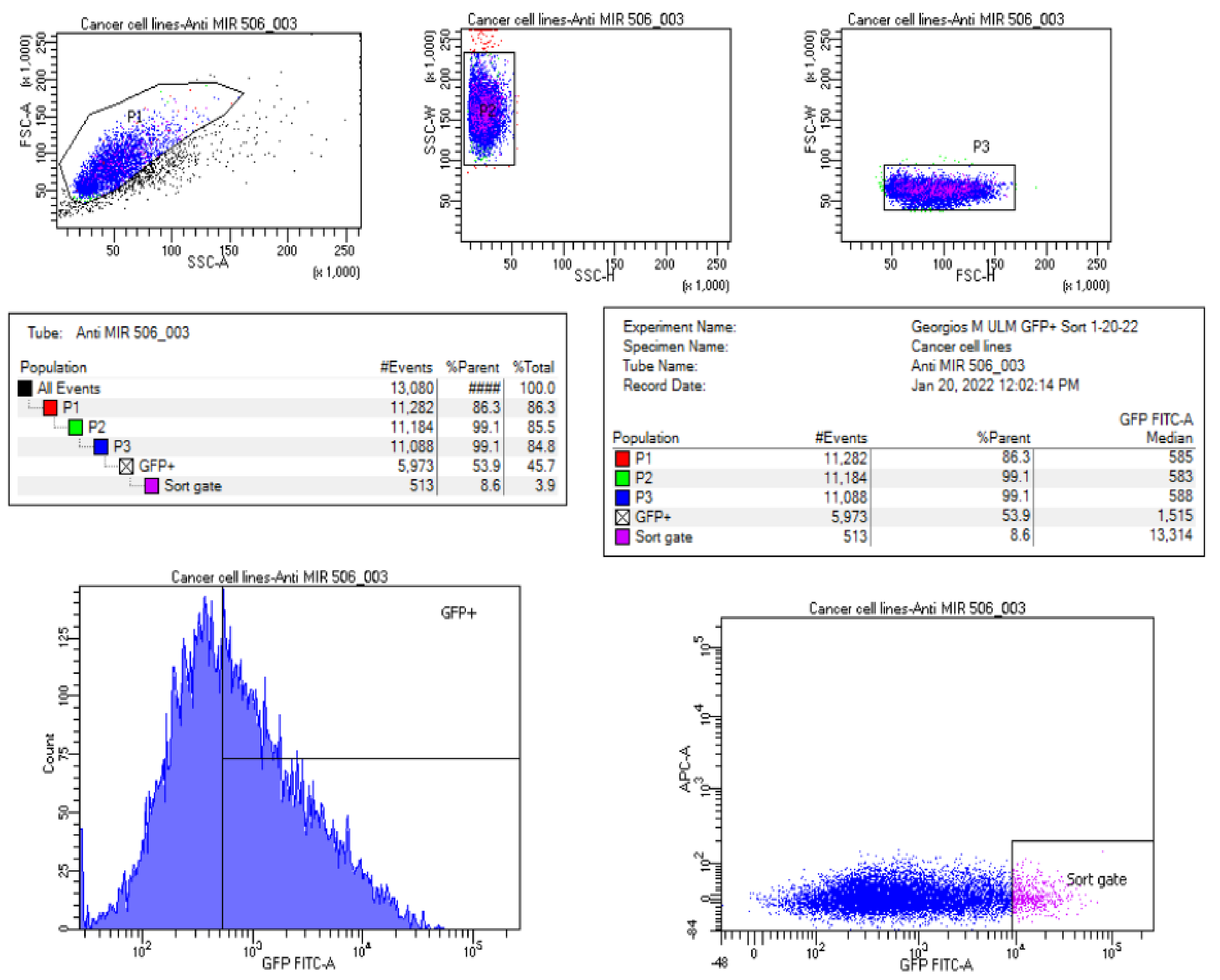

Sorting analysis of A549-miR-143/506 cells

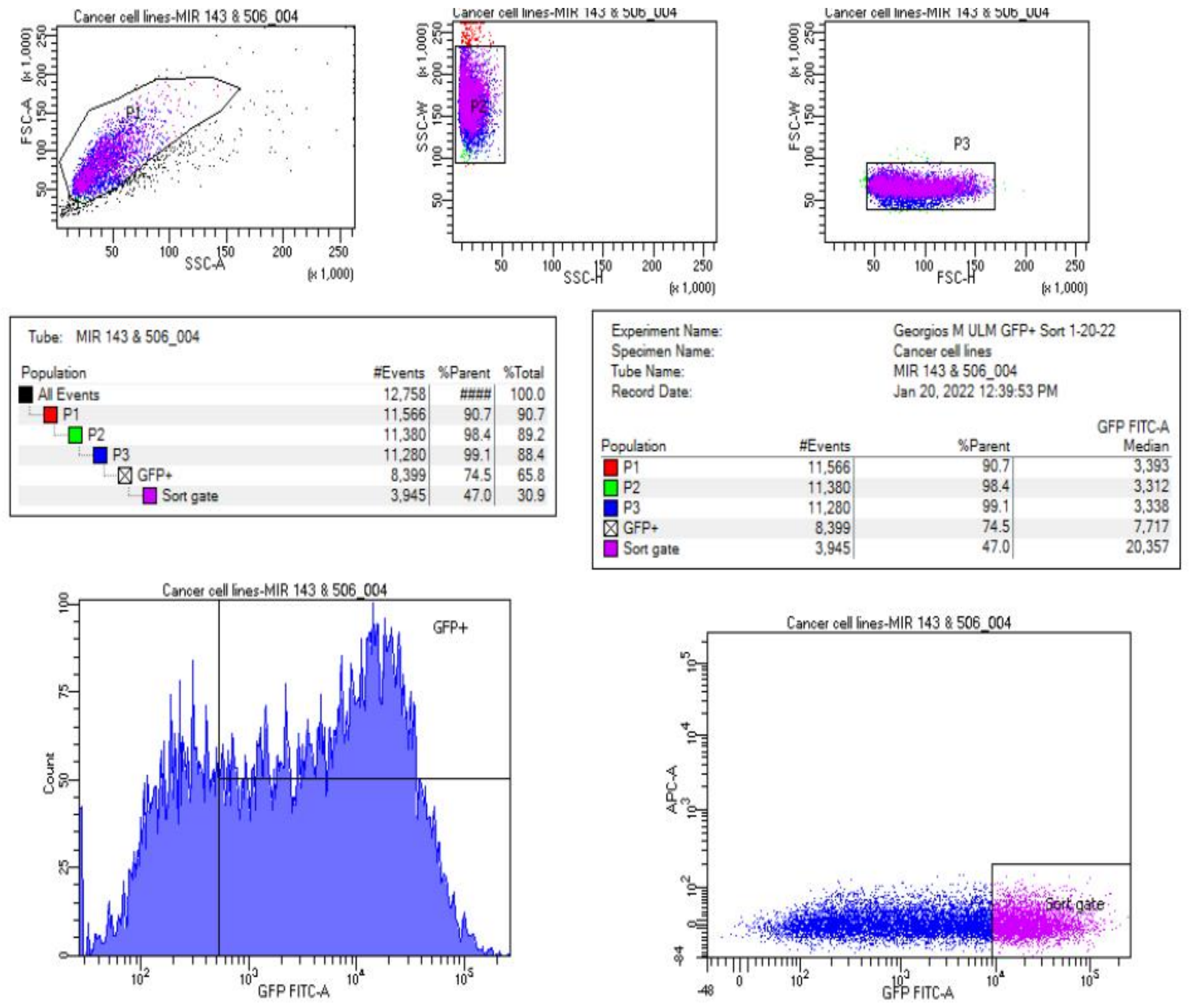

Sorting analysis of A549-anti-miR-143/506 cells

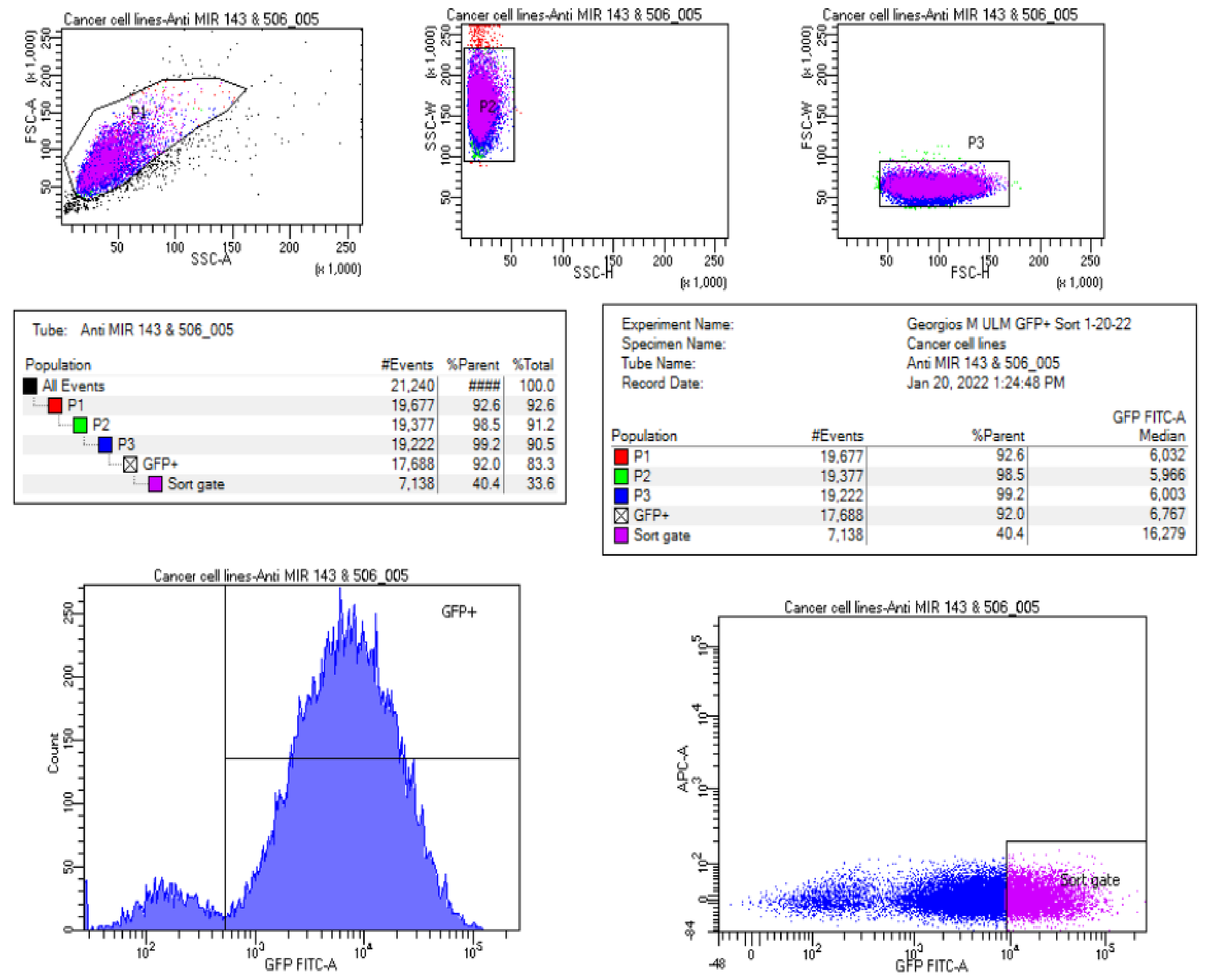

Supplementary Figure S3: Cell authentication of A549 transduced cells for the different miR deregulations indicated 100% match to the parent A549 cell line.

% Match	ATCC® Cat. No.	Designation	D5S818	D13S317	D7S820	D16S539	vWA	TH01	AMEL	TPOX	CSF1PO
100	STRC2013	A549(m-GFP2)	11	11	8,11	11,12	14	8,9.3	X,Y	8,11	10,12
100	CCL-185	A-549; Lung Carcinoma; Human (Homo sapiens)	11	11	8,11	11,12	14	8,9.3	X,Y	8,11	10,12

% Match	ATCC® Cat. No.	Designation	D5S818	D13S317	D7S820	D16S539	vWA	TH01	AMEL	TPOX	CSF1PO
100	STRC2014	A549(ANTI_m-143)	11	11	8,11	11,12,13	14	8,9.3	X,Y	8,11	10,12
100	CCL-185	A-549; Lung Carcinoma; Human (Homo sapiens)	11	11	8,11	11,12	14	8,9.3	X,Y	8,11	10,12

% Match	ATCC® Cat. No.	Designation	D5S818	D13S317	D7S820	D16S539	vWA	TH01	AMEL	TPOX	CSF1PO
100	STRC2015	A549(m-143)	11	11	8,11	11,12	14	8,9.3	X,Y	8,11	10,12
100	CCL-185	A-549; Lung Carcinoma; Human (Homo sapiens)	11	11	8,11	11,12	14	8,9.3	X,Y	8,11	10,12

% Match	ATCC® Cat. No.	Designation	D5S818	D13S317	D7S820	D16S539	vWA	TH01	AMEL	TPOX	CSF1PO
100	STRC2016	A549(m-506)	11	11	8,11	11,12	14	8,9.3	X,Y	8,11	10,12
100	CCL-185	A-549; Lung Carcinoma; Human (Homo sapiens)	11	11	8,11	11,12	14	8,9.3	X,Y	8,11	10,12

% Match	ATCC® Cat. No.	Designation	D5S818	D13S317	D7S820	D16S539	vWA	TH01	AMEL	TPOX	CSF1PO
100	STRC2017	A549 (ANTI_m-506)	11	11	8,11	11,12	14	8,9.3	X,Y	8,11	10,12
100	CCL-185	A-549; Lung Carcinoma; Human (Homo sapiens)	11	11	8,11	11,12	14	8,9.3	X,Y	8,11	10,12

% Match	ATCC® Cat. No.	Designation	D5S818	D13S317	D7S820	D16S539	vWA	TH01	AMEL	TPOX	CSF1PO
100	STRC2018	A549 (m-143/506)	11	11	8,11	11,12	14	8,9.3	X,Y	8,11	10,12
100	CCL-185	A-549; Lung Carcinoma; Human (Homo sapiens)	11	11	8,11	11,12	14	8,9.3	X,Y	8,11	10,12

% Match	ATCC® Cat. No.	Designation	D5S818	D13S317	D7S820	D16S539	vWA	TH01	AMEL	TPOX	CSF1PO
100	STRC2019	A549 (ANTI_m-143/506)	11	11	8,11	11,12	14	8,9.3	X,Y	8,11	10,12
100	CCL-185	A-549; Lung Carcinoma; Human (Homo sapiens)	11	11	8,11	11,12	14	8,9.3	X,Y	8,11	10,12

Supplementary Figure S4: Mycoplasma analysis of transduced A549 cells with the various miR deregulations. PC: positive control

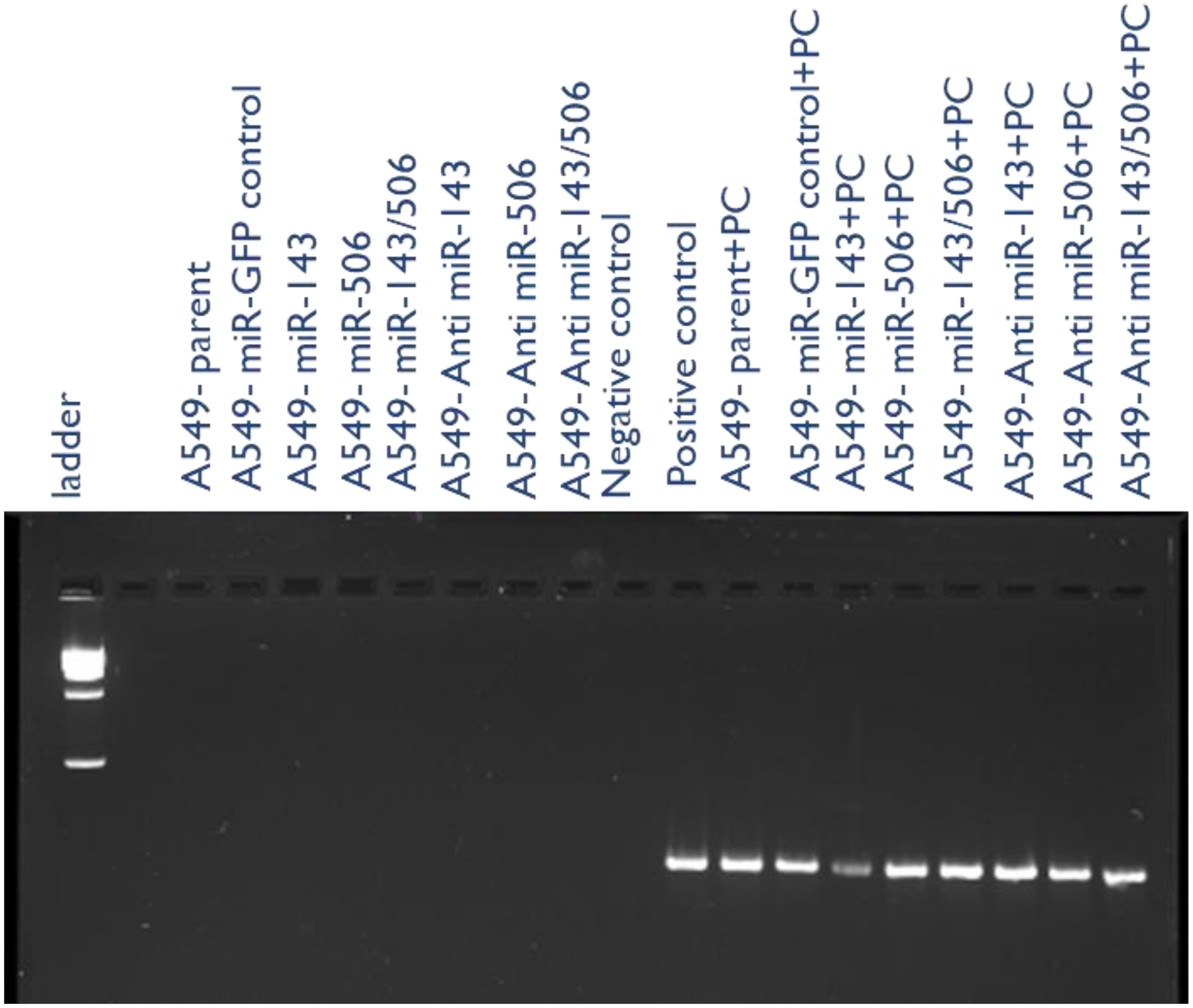

Supplementary Figure S5: Representative cell cycle analyses images for the different miR deregulated A549 cells using ModFit LT 5.0.

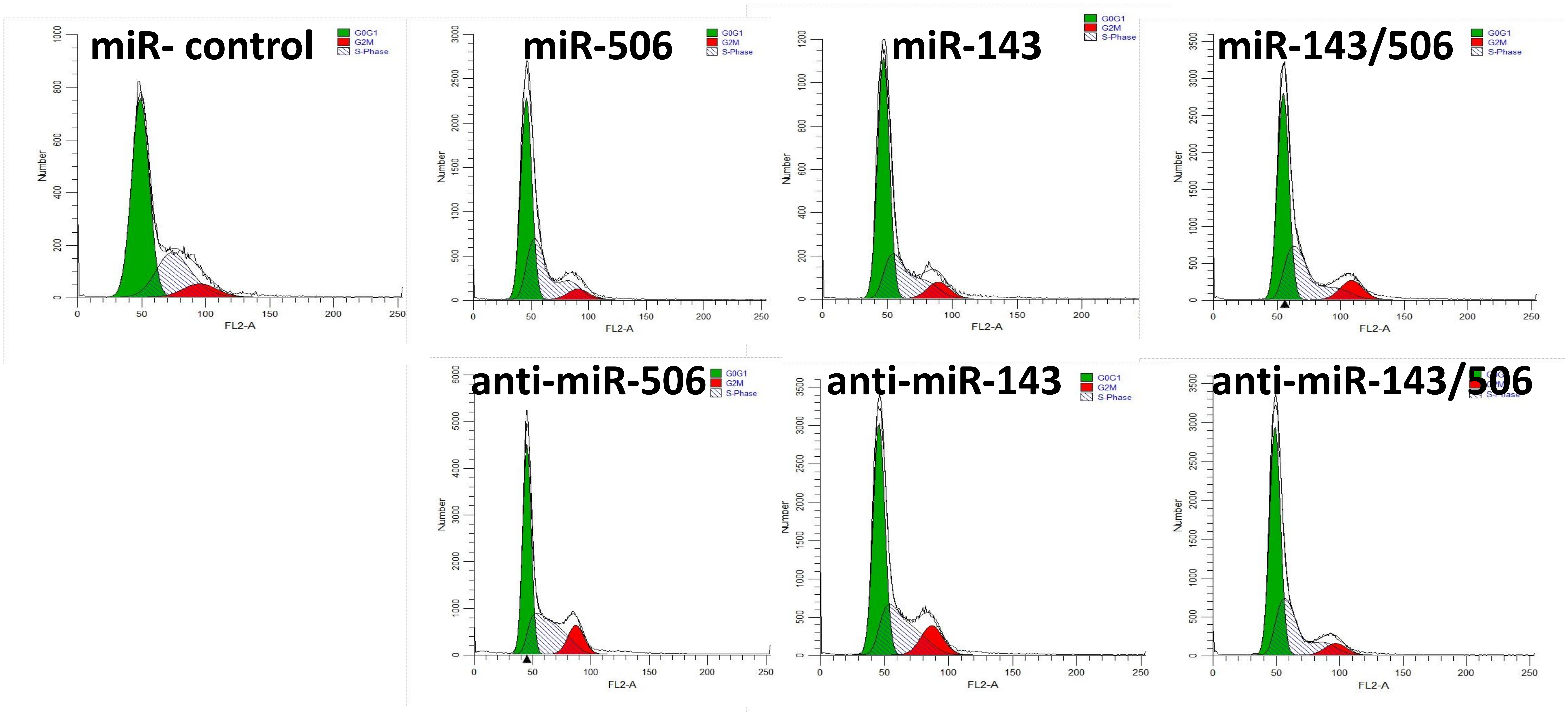

Supplement: Supplementary file 1 [file ijms-25-04432-s001.zip › Supplementary file1.pdf]
